# Achieving Health Equity Through Community Engagement in Translating Evidence to Policy: The San Francisco Health Improvement Partnership, 2010–2016

**DOI:** 10.5888/pcd14.160469

**Published:** 2017-03-23

**Authors:** Kevin Grumbach, Roberto A. Vargas, Paula Fleisher, Tomás J. Aragón, Lisa Chung, Colleen Chawla, Abbie Yant, Estela R. Garcia, Amor Santiago, Perry L. Lang, Paula Jones, Wylie Liu, Laura A. Schmidt

**Affiliations:** 1 Community Engagement and Health Policy Program, Clinical and Translational Science Institute, University of California, San Francisco, California; 2 Department of Family and Community Medicine, University of California, San Francisco, California; 3 San Francisco Department of Public Health, San Francisco, California; 4 Division of Oral Epidemiology and Public Health, School of Dentistry, University of California, San Francisco, California; 5 Dignity Health, Saint Francis Memorial Hospital, San Francisco, California; 6 Instituto Familiar de la Raza, Inc., San Francisco, California; 7 Chicano/Latino/Indígena Health Equity Coalition, San Francisco, California; 8 APA Family Support Services, San Francisco, California; 9 API Health Parity Council, San Francisco, California; 10 Rafiki Wellness, San Francisco, California; 11 African American Community Health Equity Council, San Francisco, California; 12 Philip R. Lee Institute for Health Policy Studies, University of California, San Francisco

## Abstract

**Background:**

The San Francisco Health Improvement Partnership (SFHIP) promotes health equity by using a novel collective impact model that blends community engagement with evidence-to-policy translational science. The model involves diverse stakeholders, including ethnic-based community health equity coalitions, the local public health department, hospitals and health systems, a health sciences university, a school district, the faith community, and others sectors.

**Community Context:**

We report on 3 SFHIP prevention initiatives: reducing consumption of sugar sweetened beverages (SSBs), regulating retail alcohol sales, and eliminating disparities in children’s oral health.

**Methods:**

SFHIP is governed by a steering committee. Partnership working groups for each initiative collaborate to 1) develop and implement action plans emphasizing feasible, scalable, translational-science–informed interventions and 2) consider sustainability early in the planning process by including policy and structural interventions.

**Outcome:**

Through SFHIP’s efforts, San Francisco enacted ordinances regulating sale and advertising of SSBs and a ballot measure establishing a soda tax. Most San Francisco hospitals implemented or committed to implementing healthy-beverage policies that prohibited serving or selling SSBs. SFHIP helped prevent Starbucks and Taco Bell from receiving alcohol licenses in San Francisco and helped prevent state authorization of sale of powdered alcohol. SFHIP increased the number of primary care clinics providing fluoride varnish at routine well-child visits from 3 to 14 and acquired a state waiver to allow dental clinics to be paid for dental services delivered in schools.

**Interpretation:**

The SFHIP model of collective impact emphasizing community engagement and policy change accomplished many of its intermediate goals to create an environment promoting health and health equity.

## Background

The San Francisco Health Improvement Partnership (SFHIP) was established in 2010 to promote health equity by using a novel collective impact model (1) that blends community engagement with policy change. Frieden’s framework pyramid for public health impact considers high impact interventions to include policy and structural interventions (2). The SFHIP model uses community engagement and evidence-to-policy population health translational science (3,4) as catalysts for high-impact community prevention initiatives (Table 1). Community engagement not only promotes participatory research and common agendas in planning and implementing health initiatives; it also unites and empowers local constituencies to seek policy changes that reduce structural inequities (5). Evidence-based policy measures, in turn, create changes in regulations and new funding streams that can sustain initiatives. SFHIP used this approach to engage diverse local health stakeholders, including ethnic-based community health equity coalitions, the public health department, hospitals and health systems, a health sciences university, a school district, the faith community, philanthropic groups, and other sectors. This article reports the process and accomplishments of 3 SFHIP initiatives: reducing sugar sweetened beverage (SSB) consumption, regulating retail alcohol sales to reduce alcohol-related violence, and eliminating disparities in children’s oral health.

**Table 1:** Principles of Collective Impact and Evidence-to-Policy Population Health Translational Science, San Francisco Health Improvement Partnership, 2010–2016

Principle	Component
Core elements of collective impact^a^	Common agenda
	Shared measurement strategy
	Mutually reinforcing activities
	Continuous communication
	“Backbone” support from organizations that provide staff and financial resources
Key ingredients of population health translational science^b^	Research responsive to user needs
	Understanding of the decision-making environment
	Effective stakeholder engagement
	Strategic communication
Health equity^c^	Achievement of social justice in health, measured by elimination of health disparities

a Source: Hanleybrown, et al (1).

b Source: Woolf, et al (3).

c Source: Braveman (5).

## Community Context

San Francisco is a county and city under unitary governance, with an ethnically diverse population of about 850,000 residents. It has many health sector assets, including a well-regarded local public health department, a world-renowned health sciences university (the University of California, San Francisco [UCSF]), highly rated hospitals and health systems, and robust community organizations. Nonetheless, San Francisco has prominent health disparities. For example, hospitalization rates for diabetes are 7 times higher for African Americans and twice as high for Latinos than for whites.

We report on policy and community engagement objectives and how they were addressed. Although each of the 3 initiatives specified its goals for population health outcomes, initiatives set their initial sights on intermediate policy objectives that had strong evidence-based links with desired health outcomes. For example, research shows that soda taxes result in reduced consumption of SSBs (6); lower consumption of SSBs is associated with lower prevalence of diabetes, dental decay, and other diseases (7). Influencing enactment of policies and ordinances, such as a soda tax, were measures of short-term success, with a logic model “connecting the dots” to longer-term good health-related outcomes.

## Methods

SFHIP evolved in 2 stages: the 2010 to 2013 phase (SFHIP 1.0) and the 2013-to-present phase (SFHIP 2.0). SFHIP 1.0 grew out of discussions among leaders of the UCSF Clinical and Translational Science Institute’s (CTSI’s) Community Engagement and Health Policy Program, the San Francisco Department of Public Health (SFDPH), and ethnic-based community health equity coalitions seeking to better align community-engaged translational science and public health practice to improve health equity. A steering committee was formed to govern SFHIP 1.0. A local scoping exercise assessed community health needs and existing improvement efforts to identify community health topics for initial projects by using the following criteria to prioritize topics: level of population health importance (based on data and community-identified needs), level of health inequity across population groups, amenability of the identified need to evidence-based preventive interventions, alignment of the need with research expertise at UCSF, and level of opportunity to build a new collective public health initiative or enhance an existing one. This exercise led SFHIP to prioritize 3 initiatives: obesity-related disease, alcohol-related violence, and children’s oral health. Other important health issues, such as HIV infection and tobacco use, were not included on the priority list because they already had high-performing, collaborative programs in place.

Partnership working groups (PWGs) were formed for each initiative; they consisted of diverse members committed to collaborating on developing and implementing action plans to “move the dial” on their respective health issue. PWGs emphasized engaging community organizations and members. At least one faculty member from UCSF with relevant expertise participated in each PWG. PWGs were encouraged to focus on feasible, scalable, and sustainable evidence-based interventions, especially policy and structural interventions. Community members participated in developing logic models for each PWG and in delineating inputs, activities, outputs, policy outcomes, and the health impact of each. Logic models were vetted at an SFHIP summit meeting in 2013, which was attended by more than 150 community members and representatives of stakeholder organization. The UCSF CTSI served as the initial backbone institution, organizing steering committee meetings, providing staff navigators, and providing small seed grants to support each PWG.

In 2013, SFHIP 1.0 joined with 2 other health improvement groups to create SFHIP 2.0. One group, Building a Healthier San Francisco, is a coalition formed by the San Francisco Hospital Council in 1994 to assess community health needs as required of nonprofit hospitals by state and now federal law. This coalition administered a comprehensive, online data repository of local population health indicators. The second group arose from an ordinance requiring a health care services master plan and SFDPH’s decision to seek accreditation by the Public Health Accreditation Board, which required an assessment of community health needs and an improvement plan (CHIP). In 2013, SFHIP 1.0, Building a Healthier San Francisco, and the CHIP group merged to form SFHIP 2.0. SFHIP.org became the data-tracking platform, and CHIP became the population health strategic plan; existing SFHIP 1.0 projects continued and new projects were launched. The SFHIP steering committee expanded its membership, and the San Francisco Hospital Council and SFDPH joined the UCSF CTSI as backbone organizations.

## Outcome

### Sugar-sweetened beverages 

#### Community context

SSBs account for 36% of the added dietary sugar consumed in the United States. SSB consumption is associated with obesity, heart and liver disease, diabetes, and dental decay (7). In San Francisco, 34% of African American and 24% of Latino children and adolescents consume 2 or more sugary beverages per day, compared with 4% of whites (8). Research documents the effectiveness of regulatory and tax policies in reducing consumption of SSBs (6).

#### Methods

In 2006, then-Mayor Gavin Newsom launched the Shape UP San Francisco coalition to promote environments supporting healthy eating and active living. The SFHIP PWG on obesity began by augmenting the efforts of Shape UP San Francisco on several projects. With time, the PWG decided to concentrate its effort on policy and educational interventions to reduce SSB consumption (Table 2). UCSF researchers collaborated with navigators, SFDPH staff members, and health advocates to brief San Francisco policymakers on the science demonstrating adverse health outcomes of SSB consumption and the evidence of effectiveness of regulatory and pricing policies in reducing consumption. SFHIP institutional members also considered how they might implement their own internal SSB policies.

**Table 2:** Characteristics and Outcomes of Three Initiatives, San Francisco Health Improvement Partnership, 2010–2016

Initiative Component	Outcome
**Sugar-Sweetened Beverage (SSB) Initiative**	
**Objective**	To implement public and private policies to reduce consumption of sugary beverages
**Key stakeholder participants**	Shape Up San Francisco coalition; University of California San Francisco (UCSF) Clinical and Translational Science Institute (CTSI); San Francisco Board of Supervisors, San Francisco Department of Public Health (SFDPH); Public Utilities Commission; Chicano/Latino/Indigena Health Equity Coalition; African American Community Health Equity Council; Asian and Pacific Islander Health Parity Coalition; community hospitals; San Francisco Unified School District (SFUSD).
**Achievements**	Most San Francisco hospitals implemented or have committed to implementing healthy beverage policies prohibiting serving or sale of SSBs (2015–2017). 2 new local SSB ordinances were enacted banning use of San Francisco government funds to purchase SSBs and requiring health warnings on advertisements for SSBs (2015). Sugar tax ballot measures were qualified for June 2014 and November 2016 elections; June 2014 ballot measure was defeated by voters but voters passed the November 2016 ballot measure. 19 new neighborhood tap water filling stations installed in low-income neighborhoods (2016–2017). SFUSD wellness policy was adopted prohibiting sale or serving of SSBs (2016) 9 community health workers were trained on SSBs for education campaign in low income and minority neighborhoods (2016).
**Alcohol Policy Initiative**	
**Objective**	To strengthen implementation and enforcement of regulation of retail alcohol sales to increase neighborhood safety.
**Key stakeholder participants**	San Francisco Alcohol Prevention Coalition, UCSF CTSI, SFDPH, San Francisco Police Department ABC Liaison Unit, community organizations, health equity coalitions, neighborhood economic development organizations, DataKind, and the San Francisco Brigade of Code for America.
**Achievements**	San Francisco Board of Supervisors issued a policy statement recommending a state ban on the sale of powdered alcohol; the California State Legislature subsequently enacted a ban (2015–2016). San Francisco Board of Supervisors passed a resolution calling on the California State Alcoholic Beverage Commission not to issue alcohol licenses to formula retail businesses (Starbucks, Taco Bell) in San Francisco; as a result, the businesses subsequently withdrew their license applications (2015–2016). San Francisco Health Improvement Partnership (SFHIP) Alcohol Policy Partnership Working Group established and supported community alcohol policy *promotoras* to build community capacity to engage in policy implementation, monitoring, and enforcement (2015–2017).
**Children’s Oral Health Initiative **	
**Objective**	To develop and implement a city-wide strategic plan to reduce disparities in children’s oral health.
**Key stakeholder participants**	SFDPH’s oral health division and primary care clinics, UCSF CTSI and School of Dentistry, University of the Pacific School of Dentistry, San Francisco Community Clinic Consortium and its Federally Qualified Community Health Centers, health equity coalitions, San Francisco Dental Society, SFUSD, Head Start.
**Achievements**	Developed a citywide strategic plan written by SFHIP partnership working group and adopted by the San Francisco Health Commission (2013–2015). Created a new SFDPH position, Children’s Oral Health Coordinator, with city funding (2016). Launched the community-driven Chinatown Children’s Oral Health Task Force, acquiring $250,000 in new city funding for several neighborhood task forces (2016). Trained more than 70 primary care medical providers to apply fluoride varnish to children’s teeth (2013–2016). Increased the number of clinics providing fluoride varnish at routine well-child visits from 3 to 14 (2013–2017). Acquired a waiver from the California Department of Education to allow dental clinics to be paid for dental services delivered in schools, paving the way for local community dental clinics to begin providing sealants and other preventive dental services in San Francisco schools (2016).

Community engagement was essential for incorporating a community voice in policy deliberations and mobilizing residents to advocate for policy changes. PWG partners conducted a community participatory research project that used focus groups to explore attitudes toward SSB regulatory and tax policies among residents of low-income neighborhoods — communities most affected by SSB intake and heavily targeted by SSB companies’ marketing. Community members collaborated with UCSF and SFDPH personnel on all aspects of the study, and the ethnic health equity coalitions served as conveners for the focus groups. The study found that misgivings about a soda tax were partly mitigated when residents had confidence that government would spend the funds on public health programs benefiting their community and that there would be greater availability of free, clean drinking water (eg, neighborhood bottle-filling stations) as an alternative to SSBs.

#### Outcomes

Public policy outcomes include the Board of Supervisors enacting ordinances prohibiting purchase of SSBs with San Francisco government funds and requiring beverage companies to place labels on SSB advertisements in San Francisco warning consumers of their health risk. The Board of Supervisors also placed ballot measures to enact a soda tax before the electorate in 2014 (did not pass) and in November 2016 (did pass). Informed by the community-based participatory research study highlighting the desire of community members for better access to clean tap water in low-income communities, SFHIP worked with the San Francisco Public Utilities Commission to deploy new filtered tap-water filling stations at libraries, parks, and other public venues in low-income neighborhoods.

Institutional members also implemented SSB policies. UCSF adopted a campus-wide policy eliminating SSBs from patient menus, cafeterias, retail food outlets, and vending machines. Kaiser Permanente adopted a comparable policy, and the 2 other large hospital systems in San Francisco are moving to implement healthy beverage policies. The county hospital falls under the SSB purchase prohibition of the local ordinance. San Francisco is poised to be the first city in the United States to have virtually all its hospitals prohibit distribution or sale of SSBs. The San Francisco Unified School District, which already had banned sale of SSBs on school premises, adopted a wellness policy with stringent restrictions on serving of SSBs at school events.

### Alcohol

#### Community context

Alcohol-related premature mortality accounts for about 10% of all years of life lost among men in San Francisco, with prominent disparities among African Americans and Latinos (9). Areas highly saturated with alcohol retail outlets experience high rates of alcohol-related health and safety problems (10,11). San Francisco has the greatest density of alcohol retail outlets of any city in California: 75 outlets per square mile compared with an average of 10 per square mile for all cities in the state.

#### Methods

The SFHIP Alcohol Policy Partnership Working Group (APPWG) was organized to reduce disparities in alcohol-related harm, particularly violence and public nuisance activities (Table 2). APPWG developed a diverse multisector partnership spearheaded by community members living and working in neighborhoods burdened by a large retail alcohol footprint and high rates of alcohol-related health and safety problems; the partnership was developed in collaboration with SFDPH, the San Francisco Police Department, and other stakeholders. APPWG emphasizes environmental solutions, focusing on local policies that regulate availability of alcohol and set community norms regarding alcohol’s appeal and economic benefit.

The group developed data tools and conducted applied research to inform community organizing and policymaking. With donated support from 2 technology industry nonprofit organizations, the APPWG developed a tool for mapping alcohol outlets. This interactive tool incorporates geographic data on alcohol outlets, crime rates, population demographics, and incidence of alcohol-related harms in order to illustrate graphically how the city’s alcohol environment is associated with health disparities. The tool was valuable for the group’s community capacity building and for engagement efforts with policy makers, residents of the most affected neighborhoods, and other partners. Collaboration between APPWG and the San Francisco Injury Center resulted in the Zuckerberg San Francisco General Hospital and Trauma Center’s starting a program to test patients’ blood alcohol routinely. The hospital followed with a study using data on blood alcohol levels and APPWG’s mapping tool that found that alcohol outlet density is a strong predictor of injuries involving patients with high blood alcohol levels (12).

APPWG policy efforts focused on strengthening San Francisco’s Deemed Approved Uses Ordinance (DAO) regulating retail alcohol outlets, including requiring public health assessments with active community participation when new alcohol licenses are under consideration for approval. DAO reforms were hampered by the complexity of the ordinance, difficulty coordinating multiple regulatory offices, and interest-group resistance to change. Community focus groups conducted under the auspices of SFHIP in the most alcohol outlet-dense areas of San Francisco confirmed that residents find it difficult to understand and participate in DAO implementation and enforcement. APPWG launched a community education campaign, starting with leaders of local community organizations and expanding into a policy *promotoras *(Hispanic outreach worker) program to train community members to educate and empower one another about the ordinance.

#### Outcomes

Armed with its research studies and mapping tools, APPWG provided a quick-response team to address urgent concerns about local alcohol retail issues, such as the emergence of powdered alcohol as a retail product and alcohol licensing of chain stores. APPWG’s statement opposing the sale of powdered alcohol was quickly ratified by SFHIP and influenced enactment of a state law banning sale of powdered alcohol. When Starbucks and Taco Bell sought liquor licenses for their San Francisco franchises, community partners brought this to the attention of APPWG, which worked with the Board of Supervisors to pass a unanimous resolution urging the state not to issue licenses in San Francisco to this class of retailer. Starbucks and Taco Bell subsequently withdrew their license applications. Another outcome was that SFDPH convened the DAO regulatory partners to analyze the ordinance and recommend changes.

### Children’s oral health

#### Community context

Dental caries is the most common childhood chronic disease (13). Despite fluoridation of San Francisco’s water system, 35% of children enrolled in San Francisco public schools have dental decay by the time they enter kindergarten. Latino, African American, and Chinese American kindergartners in San Francisco are 2 to 3 times more likely than their white counterparts to have untreated dental caries (14). In addition to fluoridated water, good oral hygiene practices (including avoiding SSB consumption) and regular dental care can prevent caries. Access to dental care is particularly challenging for children from low-income families; most private dentists in San Francisco do not participate in Medicaid, and there is limited oral health professional capacity in safety net dental clinics and school-based settings. Application of topical fluoride varnish by medical personnel during well-child visits is efficacious in reducing rates of caries (15), providing evidence for mainstreaming this practice into routine well-child primary care visits.

#### Methods

The SFHIP Children’s Oral Health Working Group (COHWG) was formed to address disparities in prevalence of childhood caries (Table 2). In consultation with community stakeholders and public health leaders, COHWG decided that the first step was to align sectors under a coordinated, community-driven strategic plan and obtained funding from a local foundation to support the planning process. The COHWG steering committee presented its comprehensive assessment of the landscape of San Francisco children’s oral health at a community retreat attended by more than 50 people representing diverse stakeholders. Workgroups were formed in 4 action areas — promotion, access to dental care, integrating oral health into overall health, and evaluation — and tasked with developing action plans and measurable outcomes. An overall vision statement was created: “All children in San Francisco are caries-free.” The 1-year development of the strategic plan culminated in the San Francisco Health Commission adopting the plan as the official citywide blueprint for action, an important step for engaging local policymakers and raising awareness about the public health issues related to children’s oral health.

Planning work groups transitioned into implementation teams supported by a grant from another local foundation. Following a community briefing on high caries prevalence among Chinatown children, community organizations in Chinatown mobilized to form a neighborhood children’s oral health task force that has become a leader in educational and advocacy work and has served as a model for developing task forces in other high-prevalence neighborhoods.

#### Outcome

Policy accomplishments include the San Francisco government establishing and funding a permanent oral health coordinator position in the SFDPH to coordinate the implementation of the strategic plan and the dissemination of $250,000, which was appropriated to fund neighborhood task forces on children’s oral health. The San Francisco Health Plan, the major local Medicaidmanaged-care plan, included in its performance improvement plan a financial incentive rewarding primary care clinics that administer fluoride varnish at well-child visits. Nearly 400 additional fifth and sixth graders were added to the school sealant program, and COHWG focused on qualifying preventive services provided by dental providers in schools and other nonclinic settings for Medicaid payment.

At the inception of COHWG, the only medical providers routinely administering fluoride varnish were the SFDPH pediatric clinic at the county hospital and some pediatric practices at San Francisco Kaiser Permanente. Four additional primary care clinics in SFDPH and 2 Federally Qualified Health Centers are now providing fluoride varnish. SFDPH and UCSF staffs trained more than 70 medical staff members at these clinics to administer fluoride varnish.

## Interpretation

SFHIP and its working groups succeeded in helping to implement many of the policies and programs specified as key intermediate objectives. Many important groups operating outside SFHIP also played a role in advancing these goals; therefore, progress should not be considered entirely attributable to SFHIP.

SFHIP demonstrates the value of a collective impact approach (ie, an approach that combines community engagement with evidence-to-policy translation). Diverse stakeholders aligned around a shared agenda on health improvement initiatives. Emphasis on evidence to policy integrated translational science with public health practice, which contributes to implementation of policy and structural changes that fall in the high-yield zone of the public health impact pyramid. Examples of organizations involved in SFHIP’s collective impact approach are nonprofit hospitals that implement SSB policies congruent with local government SSB regulations, academic researchers partnering with the local health department and community organizations on participatory translational research to inform strategic SFHIP action plans, and community members advocating for SSB policies and for increased local government funding for neighborhood task forces for children’s oral health. An example of the importance of community members being directly involved is the role that residents of low income neighborhoods had in showing that policies such as a soda tax to discourage SSB consumption need to be coupled with policies to promote access to free, clean tap water for these policies to gain broader community support. Listening to the community voice following the defeat of the 2014 soda tax ballot explains in part the success of the 2016 ballot measure.

Collaborative leadership and backbone resources were critical. Individuals participating in the steering committee and PWGs brought passionate commitment to health equity, tempered by willingness to invest in building trusting relationships and consensus decision-making. Many participants, especially community representatives, volunteered much time outside the scope of their compensated jobs. The SFHIP staff members who were contributed in-kind by the backbone organizations were invaluable. Particularly vital was the skill of SFHIP staff in navigating the different cultures of government agencies, community groups, universities, and other partners to build trust and common purpose. Grant funding from local foundations supported initiatives, and UCSF’s National Institutes of Health Clinical and Translational Science Award funded UCSF faculty and staff effort.

SFHIP also experienced challenges. Building trust required overcoming a history of strained relationships among some of the partners and concerns about large institutional partners exerting excessive influence. It was not always easy for partners to relinquish individual agendas to forge a common one, whether it was a UCSF faculty member’s personal research agenda, a hospital’s traditional community benefit orientation, or a community organization’s programming comfort zone. Partners underestimated the time and effort required for cohesive group formation in the early stages of establishing a steering committee and setting priorities. SFHIP confronted the tension of policymaking invariably involving politics. Resistance from commercial interest groups impeded some SFHIP policy goals.

Another tension was over measuring success on the basis of achieving intermediate policy goals. Some stakeholders and funders were impatient to see evidence of improved public health indicators and health equity. Such outcome-oriented evaluations are limited by the time needed for changes to materialize, and the cost of performing rigorous outcome evaluations. In areas where SFHIP projects mustered the ability to evaluate rigorously, results are encouraging; for example, evaluation of implementation of the healthy beverage policy at UCSF demonstrated significant reductions in SSB consumption among lower-wage employees — the group with the highest baseline consumption.

A final tension was whether SFHIP should focus more on the first tier of the health impact pyramid: the fundamental socioeconomic determinants of health. The steering committee is still considering how SFHIP might engage in issues such as the city’s housing affordability crisis and community displacement, structural racism, and employment development.

SFHIP’s novel approach to collective impact may offer lessons for health equity initiatives in other communities. SFHIP accomplished many of its intermediate goals for aligned activities and policy change, which accomplishment augurs well for improvements in community health and health equity over time.
